# Psychosocial barriers and enablers of exclusive breastfeeding: lived experiences of mothers in low-income townships, North West Province, South Africa

**DOI:** 10.1186/s13006-020-00320-w

**Published:** 2020-08-26

**Authors:** Chantell Witten, Nicole Claasen, Herculina S. Kruger, Anna Coutsoudis, Herman Grobler

**Affiliations:** 1 Centre of Excellence for Nutrition, Faculty of Health Sciences, North-West University, Potchefstroom, South Africa; 2 Division Health Professions Education, Faculty of Health Sciences, University of the Free State, Bloemfontein, South Africa; 3 Africa Unit for Transdisciplinary Health Research, Faculty of Health Sciences, North-West University, Potchefstroom, South Africa; 4 Paediatrics & Child Health, University of Kwazulu-Natal, Durban, South Africa; 5 Community Psychosocial Research, Faculty of Health Sciences, North-West University, Potchefstroom, South Africa

**Keywords:** Exclusive breastfeeding, Psychosocial factors, Perceptions, Challengers, Mixed methods

## Abstract

**Background:**

Despite national efforts to promote exclusive breastfeeding (EBF), South Africa’s EBF rate is only 32 %. The aim of this study was to examine the rate of EBF discontinuation and the lived experiences of breastfeeding mothers at postnatal time points 3–14 days, 4–8 weeks, 10–14 weeks and 20–24 weeks.

**Methods:**

This community-based mixed-methods study collected data within a prospective cohort study on sociodemographics, the Edinburgh Postnatal Depression Scale (EPDS) and the Breastfeeding Self-Efficacy Scale-Short Form (BSES-SF) at 6–8 weeks with infant feeding data collected at 4–8, 10–14 and 20–24 weeks from 159 mothers living in low income areas. Six focus groups with 32 mothers with infants aged 6–24 weeks were conducted. Descriptive statistics was used for the quantitative data and thematic analysis for qualitative data.

**Results:**

The majority of mothers were unmarried (84.9%), living with family (69.2%) and unemployed (74.2%). Exclusive breastfeeding decreased from 34% at 4–8 weeks to 9.7% at 20–24 weeks. Mixed feeding with infant formula increased from 17.0 to 30.6% and food feeding from 3.1 to 54.2%. While there were no statistically significant associations between EBF and any of the quantitative sociodemographic variables, in the qualitative data, codes associated with barriers were more than enablers. The themes were **Mothers’ attributes (wellbeing, experiences and relationships)** with the code *mother’s stress* the strongest barrier, **Mother’s knowledge, attitudes and practices of breastfeeding** with the code *conventional medicines* the strongest barrier, **Family environment** with the code *home setting* the strongest barrier, **Social environment** with *public spaces and places* a barrier and in **Baby cues** the code *baby stomach ailments* the barrier. Within these same themes *mother’s positive emotions*, *benefits of breastfeeding*, *support in the home*, access to information and services from *health professionals* and *baby’s health* were strong enabling factors.

**Conclusions:**

Low EBF, high mixed feeding and a high EPDS score were explained by the barriers identified in the qualitative data. The data suggests that mothers from low-income households would be better supported through interventions that address food insecurity; family relationships and those that build confidence in mothers and resilience in confronting difficult and hostile breastfeeding environments.

## Background

The World Health Organization (WHO) and UNICEF [1] have set as one of the Global Nutrition targets, an exclusive breastfeeding (EBF) rate of 50% at country-level by 2025; a global effort that undisputedly will contribute towards the achievement of the Sustainable Development Goals (SDGs) [2, 3]. Until the industrial production of formula milk, human milk was needed for human survival. Despite the wide use of infant formula, recent scientific findings reaffirm and assert that no food is more nutritious for an infant than human milk. Human milk is a unique, biomedical product that is the best and most complete natural food that provides for all the infant’s physiological needs during the first 6 months of life [4–7].

While the life-saving benefits of breastfeeding have been documented over many decades, the first-ever Lancet Series on Breastfeeding in 2016 reaffirmed and put forward a call for the scale up of breastfeeding interventions towards reducing infant morbidity and mortality [8]. A number of systematic reviews on interventions in low- and middle-income countries have further shown positive outcomes for breastfeeding initiation and exclusivity to 6 months. Most of these interventions have focused on provision of breastfeeding information, peer support and breastfeeding problem-solving [9–11]. While on the other hand, qualitative studies have identified barriers that are not related to insufficient or inaccurate information or the lack of mentoring support for breastfeeding. These barriers were mostly related to milk insufficiency beliefs, cultural beliefs and practices, health systems and gender and power-relations [12–14]. A systematic review on studies from Brazil [15] reported maternal employment as the most frequently cited barrier to EBF followed by maternal perceptions of insufficient breast milk supply and medical barriers related to illness of mothers and/or infants, as well as breast problems. These barriers are unlikely to be responsive to interventions offering more breastfeeding information, or additional mentoring or peer support.

Since the 2014 global commitment by the United Nations to improve breastfeeding [16, 17], a plethora of platforms to provide breastfeeding information and support have evolved to address aspects that may particularly undermine mothers’ successful breastfeeding practices, namely, mothers’ self-confidence and psychological disposition [18, 19]; mother’s resilience to risks and threats [20, 21]; and the sociocultural practices [14, 22–24] that will require localized and tailored interventions to suit the mother’s needs and context. A systematic review identified that for low- and middle-income countries there were few intervention studies targeting mothers with infants aged 1 to 5 months that were conducted in the family or community setting and even fewer that used integrated media or social media to improve EBF outcomes [9]. Even more concerning are the lack of large-scale interventions to address these particular barriers faced by the mother in her home and family setting [25, 26].

Psychosocial factors, defined as a combination of psychological and social factors include individual-level processes and meanings that influence an individual’s mental state while social factors are general factors at the level of human society concerned with social structure and social processes that impinge on the individual [27]. Psychosocial also implies that the effect of social processes is sometimes mediated through psychological understanding. Since the breastfeeding policy shift in 2011, South Africa has made a concerted effort to improve the breastfeeding environment through policy reform [28, 29], information dissemination [30, 31] including the legislation of the International Code for the Marketing of Breast Milk Substitutes [32]. In an effort to inform South Africa’s breastfeeding communication strategy [33], the motivation for this study was to identify the point-in-time psychosocial barriers and enablers of EBF from the lived experiences of mothers with infants aged 4–24 weeks in a low-income township in the North West Province of South Africa. In this study a barrier was defined as any factor that would hinder or make it difficult for a mother to EBF and an enabler would be a factor that would help or support her to exclusively breastfeed.

## Methods

The aim of this mixed-methods study was to examine the rate of discontinuation of EBF at three specific postnatal time points, namely, 4–8 weeks, 10–14 weeks and 20–24 weeks and to explore the lived experiences of breastfeeding mothers in the sub-district of Tlokwe in the North West Province, South Africa.

### Setting

The study sites were two low-income townships, serviced by health clinics and district hospitals. These townships reflect the economic disparities of South Africa with wealthier suburbs juxtaposed against poor townships. Like most of South Africa, these townships are burdened with intersectional inequity. High unemployment, poor living conditions, high levels of violence and crime and low levels of social capital [34]. The North West Province experienced higher unemployment than the national average of 27.6% [35]. The major languages spoken are Setswana and Afrikaans with many people being bilingual [36]. Data for the infant feeding cohort study was collected between May 2018 and March 2019 and for the qualitative data between July and December 2018.

### Design

This was an exploratory descriptive study using a convergent parallel mixed methods design [37] to provide an in-depth understanding of infant feeding practices and experiences of a cohort of mothers followed prospectively from early breastfeeding period (day 3–14) through to the infant age of 20–24 weeks. The sample size of this study was calculated with the aim to have a minimum number of 12 [38] exclusively breastfeeding mothers at week 6–8 for the in-depth interviews. Based on the findings of previous studies in South Africa [39, 40], exclusive breastfeeding rates were reported to be approximately 8 and 1%, respectively, at weeks 6–8 and at week 24 therefore the sample size for this study was based on 178 participants at baseline, with an over-estimation of expected dropout rate of 10% for mortality and/or loss to follow up. We estimated to have 144 mothers at 4–8 weeks, 117 mothers at 10–14 weeks and 95 mothers at 20–24 weeks, with at least twelve, nine and one exclusively breastfeeding mother, respectively, at 4–8 weeks, 10–14 weeks and at 20–24 weeks. At all time-points, numbers of participants were close to the planned sample size, except at 20–24 weeks, where we interviewed only 72 mothers, compared to the planned sample size of 99, but this group still had more than the expected number of exclusively breastfeeding mothers.

In order to reach mothers with infants with similar ages to the cohort study participants and to avoid a biased sample from the Tlokwe sub-district, six focus groups were conducted with mothers with infants aged 6–24 weeks in the neighbouring sub-district. This neighbouring sub-district had a similar sociodemographic profile as the township in which the quantitative data was collected.

### Quantitative data collection and analysis

The prospective cohort study applied survey questionnaires which were administered face-to-face at the participant’s home or at the routine clinic visit. The questionnaire included an infant feeding and food frequency questionnaire (IFFFQ) administered at each time point, the Edinburgh Postnatal Depression Scale (EPDS), Breastfeeding Self-Efficacy Scale (BSES-SF) and the sociodemographic questionnaire each only administered once at 4–8 weeks.

### The infant feeding and food frequency questionnaire (IFFFQ)

The IFFFQ food categories are based on the validated gold standard 24-h recall infant feeding questionnaire prescribed by the World Health Organization [41]. The IFFFQ is a 7-day recall on proposed food items given at three possible frequencies from once a week, two to six times a week to every day. This method allows for a longer recall period than the previous 24-h and allows one to specifically ask about items mothers do not always consider food, such as teas; water; supplements; herbal medicines; and over-the-counter self-prescribed medicines. Goosen et al. [42] used a similar categorization of foods in their study, which particularly also included a question on non-prescribed over-the-counter medicines. In a pilot study with six postpartum mothers, no semantic differences in language or understanding were detected and there was no difficulty in the reading and comprehension of the tool.

### The Edinburgh postnatal depression scale (EPDS)

The EPDS is a 10-item questionnaire that was developed to identify women who have postpartum depression. Each item is scored 0 to 3. The overall assessment is done by total score, which is determined by adding together the scores for each of the 10 items. Scores could range from 0 to 30. Scores higher than 10 indicate presence of depressive symptoms [43]. Mokwena and Shiba [44] had previously translated and tested a Setswana version of this tool for their study in a different part of South Africa. This version was tested for comprehension in a pilot study with six postpartum mothers from the study area but not part of the cohort study. Colloquial semantic differences were detected and adjusted for the translated version to improve reading and comprehension of the tool.

### Breastfeeding self-efficacy scale (BSES-SF)

The BSES-SF is a 14-item self-reported instrument [42]. All items are presented positively using a 5-point Likert-scale where 1 indicates ‘not at all confident’ and 5 indicates ‘always confident’. A total summed score could range from 14 to 70 and the higher the score, the higher the level of breastfeeding self-efficacy. This tool was translated and validated for the study population by the research team. (Details included in a separate manuscript which has been submitted for publication).

### Sociodemographic questionnaire

A compilation of 25 questions previously asked in other studies conducted in the North West Province [45, 46]. The questionnaire covered sociodemographic background information on living arrangements, education level, relationship status, employment status, source of income, access to health information.

All quantitative data were analysed using the IBM SPSS Statistics version 25. Data with a normal distribution were expressed as means ± standard deviation (SD) and data with a skew distribution were expressed as median (25th, 75th percentiles) values. Categorical values were expressed as percentages and frequencies. The chi-square test together with Cramer’s V was used to determine associations between sociodemographic variables (age, education, employment, living arrangements, relationship status, household income and access to mHealth) and EBF. A *p* - value of less than or equal to 0.05 was considered to be statistically significant.

### Qualitative data collection and analysis

Focus Group Discussions (FGDs) were conducted with mixed groups of EBF and non-EBF mothers with infants aged 6–24 weeks. A focus group discussion guide was developed to explore two main questions, ‘What makes it difficult for a mother to only give her baby breastmilk?’ and ‘What helps a mother to only give her baby breastmilk?’. The FGDs were opened with participants’ reactions and discussion of two open source pictures depicting a happy breastfeeding mother and an anxious breastfeeding mother. Once the discussion on the pictures were exhausted, the discussion was focused specifically on the two questions to explore what are the barriers and what are the enablers of exclusive breastfeeding. Focus Group Discussions were conducted in English or in Setswana depending on the participants’ preference. All FGDs were voice-recorded, transcribed verbatim and translated into English. All identifiers were removed from the transcripts. Thematic analysis was applied by coding text and assigning codes as well as a dimensional code for barrier or enabler to each code, codes were further grouped into themes [37]. Data reduction (selecting and sorting data systematically) followed by data display (organizing and coding frequencies) using ATLAS.ti (version 8.4) was done.

## Results

**Fig. 1:**
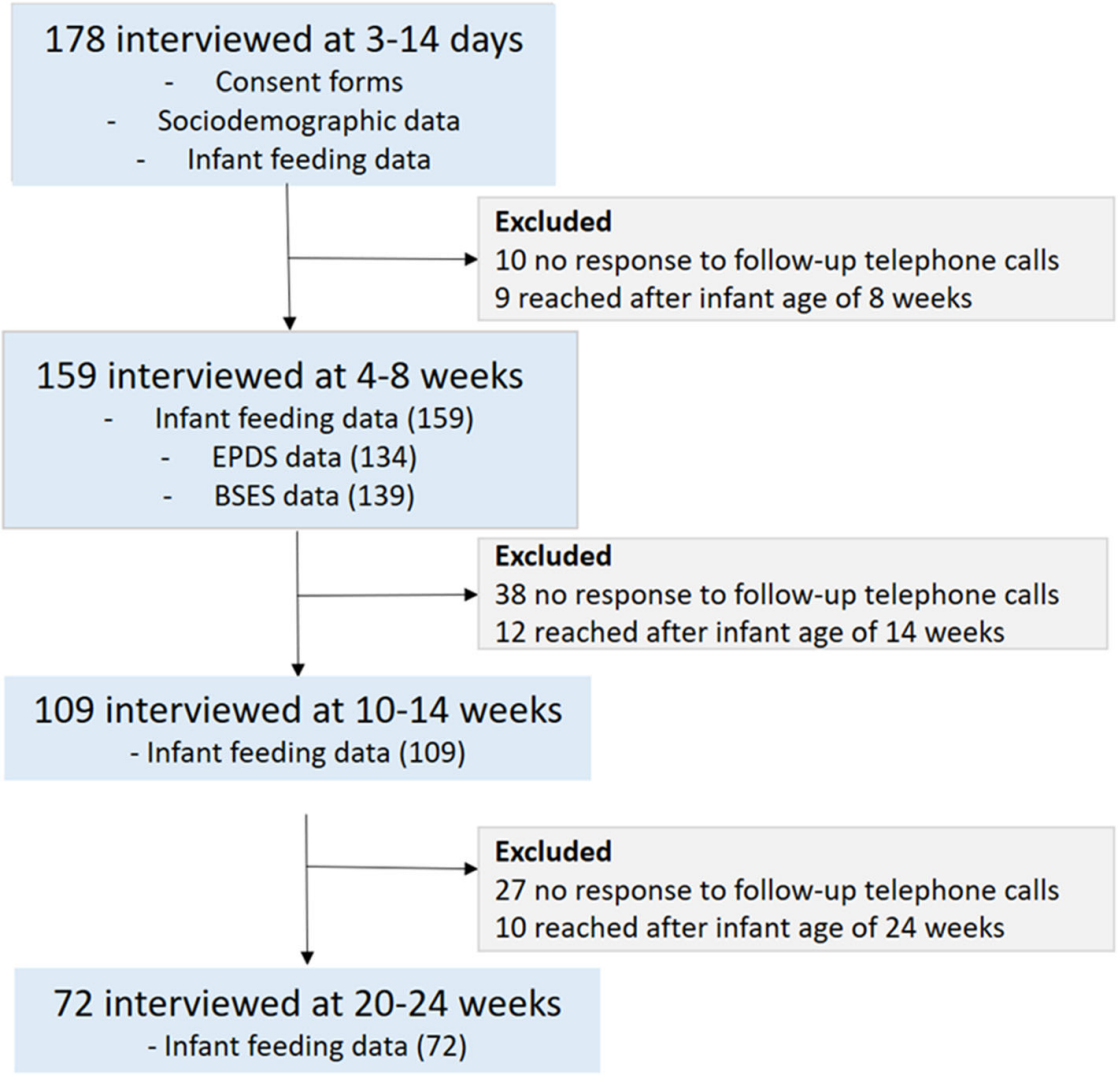
Flow diagram for the enrolment of study participants for the prospective cohort infant feeding study

The flow diagram for the recruitment and enrolment of study participants for the prospective cohort infant feeding study is shown in Fig. 1.

In a community-based study, 178 breastfeeding mothers were recruited and enrolled at 3–14 days post-partum from eight primary healthcare clinics in the Tlokwe sub-district of the North West province of South Africa. At follow-up visits at 4–8 weeks, there were 159, at 10–14 weeks, 109 and at 20–24 weeks, only 72 of the original cohort of 159 mothers. The age of participants ranged from 19 to 42 years as shown in Table 1.

**Table 1:** Sociodemographic characteristics of cohort study participants at 4–8 weeks postpartum (*n* = 159)

Sociodemographic factors	Median (interquartile range) or *n* (%)
Mother’s age (years)	27 (24, 32)
Parity	2 (1, 4)
Relationship status	
Married	24 (15.3)
Unmarried, not cohabiting	117 (73.9)
Living with a partner	18 (10.8)
Education	
Grade 0–7, primary school	12 (7.5)
Grade 8–12, high school	128 (80.5)
Post high school training	19 (12.0)
Living arrangements	
Living with family, not the father of the baby	110 (70.0)
Living with the father of the baby	46 (28.7)
Living with a new partner	3 (1.3)
Employment status	
Employed	41 (25.8)
Unemployed	118 (74.2)
Household income per month	
< R1000 (70 US dollar)	23 (14.5)
R1001-R3000 (70–200 US dollar)	43 (27.0)
R3001-R6000 (200–400 US dollar) > R6000 (> 400 US dollar)	26 (16.4) 19 (12.0)
Do not know	48 (30.1)
Edinburgh Postnatal Depression Scale (EPDS) score	
EPDS < 10	74 (55.2)
EPDS ≥10	60 (44.8)
Breastfeeding Self-Efficacy Score (BSES) - Short-Form	
BSES < 55	27 (14.4)
BSES ≥55	112 (85.6)

The cohort infant feeding patterns for infants aged 4–24 weeks are reported in Table 2.

**Table 2:** Infant feeding practices for the cohort of mothers with infants aged 4–24 weeks (*N* = 159)

Feeding practices at *N* = 159	4–8 weeks (*n* = 159) %	10–14 weeks (*n* = 109) %	20–24 weeks (*n* = 72) %
Breastfeeding	150 (94.3)	94 (86.2)	58 (80.6)
EBF^a^	54 (34.0)	32 (29.3)	13 (18.0)
Breastfeeding + non-prescribed medicines^b^	83 (52.2)	42 (38.5)	48 (66.7)
Breastfeeding + water	57 (35.8)	40 (36.7)	35 (48.6)
Breastfeeding + formula feeding (FF)	27 (17.0)	22 (20.2)	22 (30.6)
Breastfeeding + food	5 (3.1)	17 (36.7)	39 (54.2)
Formula feeding only (FF)	34 (21.4)	37 (34.0)	11 (15.3)

^a^*EBF* Exclusive breastfeeding (Breastmilk + ORS + prescribed meds only (NDOH, 2013, 2018) and ^b^Breastmilk + all medicines) (WHO, 2008 [41])

**Fig. 2:**
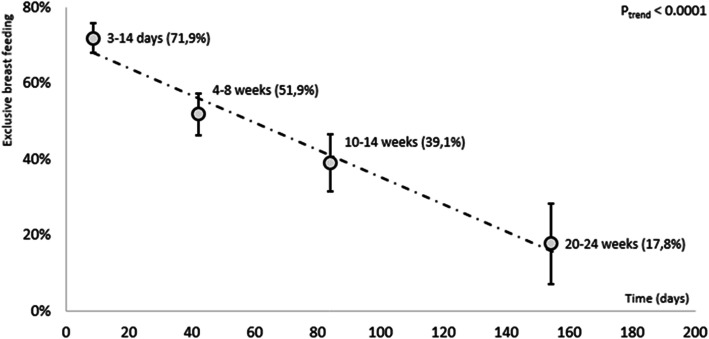
Decrease in EBF^a^ (Breastmilk + ORS + prescribed meds only (NDOH, 2013, 2018) between each time point from 3 to 4 days to 20–24 weeks

A regression line for the decrease in EBF (Breastmilk + ORS + prescribed medicines only) between time points 3–14 days to 20–24 weeks (*p* for trend < 0.0001) is shown in Fig. 2.

Associations of sociodemographic factors and EBF practices at 4–8 weeks are presented in Table 3. There were no statistically significant associations between any of the sociodemographic factors and EBF at 4–8 weeks. In Table 4 the sociodemographic and infant feeding practices data for the cohort of mothers at 20–24 weeks (*n* = 72) and the 32 mothers with infants aged 6–24 weeks in the FGDs is presented. Their sociodemographic data are similar but the infant feeding practices are significantly different given the differences in the infants’ ages. While there was no statistically significant association between any of the sociodemographic factors maternal age, parity, education, relationship status, employment status, household income, access to mHealth, EPDS or BSES-SF scores with EBF at 4–8 weeks shown in Table 3, the qualitative data provided deeper understanding of and insights into the possible reasons for observed infant feeding practices.

**Table 3:** Association of sociodemographic factors and EBF practices at 4–8 weeks

Variable	EBF^**a**^ (n)%^**#**^	Non-EBF (***n***)%	***p*** value*
Maternal age < 30 years	8 (61.5)	31 (52.5)	0.556
Maternal age ≥ 30 years	5 (38.5)	28 (47.5)	
Parity ≤2	18 (32.0)	41 (38.5)	0.985
Parity ≥3	36 (68.0)	64 (61.5)	
Education ≤ grade 12	29 (54.7)	54 (51.0)	0.383
Education > grade 12	23 (47.3)	52 (49.0)	
In a relationship Not in a relationship	46 (86.8) 7 (13.2)	94 (90.4) 11 (9.6)	0.724
Employed	14 (26.4)	28 (26.0)	0.951
Unemployed	40 (73.6)	77 (74.0)	
Household income < R3000	38 (69.8)	72 (69.2)	0.441
Household income ≥ R3000	16 (30.2)	33 (30.8)	
Receiving mHealth messages	32 (58.9)	57 (54.8)	0.660
Not receiving mHealth messages	23 (41.5)	47 (45.2)	
EPDS < 10	8 (61.5)	34 (61.8)	0.487
EPDS ≥10	5 (38.5)	21 (38.2)	
BSES < 55:	10 (0.07)	32 (23.0)	0.971
BSES ≥55:	17 (0.12)	80 (57.5)	

^a^EBF = Exclusive Breastfeeding defined as Breastmilk + ORS + prescribed meds only (NDOH, 2013, 2018)

Non-EBF = Not exclusively breastfeeding and includes breastmilk with non-prescribed medicines and/or water, and/or formula, and/or food

*EPDS* Edinburgh Postnatal Depression Score

*BSES* Breastfeeding Self-Efficacy Score

*No significant differences were found for any variables between EBF = Exclusive Breastfeeding; and non-EBF. ^#^Numbers may vary due to missing data for some variables

**Table 4:** Characteristics of the mothers in the cohort study in Tlokwe sub-district and mothers in the FGDs from the neighbouring sub-district, Matlosana

Sociodemographic data	Cohort (***n*** = 72) %	FGD women (***n*** = 32) %
Mother age range (years)	22–42	20–41
Mean age of mother (years)	28.0	30.6
Infant age range (weeks)	20–24	6–24
Unemployment status	53 (74.2)	28 (87.5)
**Infant feeding practices data**		
Breastfeeding	58 (80.6)	26 (81.2)
Exclusively breastfeeding	13 (18.0)	9 (28.1)^a^
Water giving	19 (54.2)	16 (50.0)^a^
Food feeding	43 (59.8)	7 (22.0)^a^

^a^More infants aged 6–8 weeks

The identified themes and codes from the FGDs are presented in Table 5 and are organized by frequency counts for barriers and enablers. In all themes, except Mother’s knowledge, attitudes & practices of breastfeeding the barriers were more dominant discussion points than the enablers. Table 6 presents a joint display showing the quantitative variables for EBF, mixed feeding, EPDS and BSES and the codes organized by frequency counts for barriers and enablers for each theme, **Mothers’ attributes - physical and mental wellbeing**, **experiences and relationships**, **Mother’s knowledge, attitudes and practices of breastfeeding**, **Family environment**, **Social environment** and **Baby cues** to provide insights and explanations for the poor EBF pattern observed in this cohort of mothers as shown in Table 2 and Fig. 2.

**Table 5:** Focus Group Discussion themes and codes arranged by frequency counts

Theme and codes	Barrier	Enabler
**Mothers’ attributes: physical and mental wellbeing, experience and relationships**	Frequency counts^*^	
Mother’s body image	+	+
Mother’s negative emotions (angry, unhappy)	++	–
***Mother’s positive emotions (happy, feels good)***	+	**++**^**E**^
Mother’s perception of breastmilk supply	++++	++
Mother’s experience (not first child)	+	+
Mother’s first breastfeeding experience	+	+
Mother’s health status or physical wellbeing	+++	++
Mother’s choice	+	+
Mother’s hunger & nutrition	++	++
Mother’s priorities	++	+
Mother’s sexual relationships	+	+
***Mother’s stress***	**++++**^**B**^	+
**Mothers’ knowledge, attitudes & practices of breastfeeding**		
***Benefits of breastfeeding***	–	**+++**^**E**^
Conventional medicines for babies	+++	++
Expressing breastmilk	+	+
Foods to make or increase breastmilk	+	++
Frequency and duration of breastfeeding	+	+
Information on infant feeding	+	+
***Mixed feeding***	**++++**^**B**^	+
Positioning and latching	+	+
**Family environment**		
Advice from elders	+	+
***Home setting***	**++++**^**B**^	**+++**^**E**^
Food at home	+	+
Relationship with the father of the child	+	+
**Social environment**		
***Health professionals***	++	**++**^**E**^
Breastfeeding seen as low social status	+	–
Breastfeeding in public	+	+
***Public spaces & places (malls, taxis, other people)***	**+++**^**B**^	+
Traditional beliefs & practices	+	+
Work environment	+	+
**Baby cues**		
Baby does not want to or struggles to breastfeed	+	–
Baby full	+	+
***Baby’s health***	++	**++**^**E**^
Baby not full	++	+
Baby bonding & love	+	+
Baby crying	+	+
Baby breastfeeding frequently	+	–
Baby’s growth & development	+	+
Baby sleeping longer or better	+	+
***Baby stomach ailments***	**++**^**B**^	+
Baby upset or unsettled by mother’s emotions	+	–

^*^Frequency counts based on ATLAS.ti - Fq counts^*^: 0 = −, 1–20 = +; 21–40 = ++, 41–60 = +++, > 60 = ++++

^**B**^ = Highest count for barrier and ^**E**^ = highest count for enabler

**Table 6:** Joint display of factors influencing exclusive breastfeeding in a cohort of mothers with infants aged 6–24 weeks, arranged by themes and codes with the highest frequency count by barrier (B) and enabler (E)

Socio-demographic data	Themes and codes by frequency counts for barrier (B) and enabler (E)	Interpretation of findings
There were no statistically significant associations between any of the socio-demographic factors and EBF practices.	**Mothers’ attributes: physical and mental wellbeing, experience and relationships** Mother’s stress ++++^B^ / mother’s positive emotions++^E^ **Mothers’ knowledge, attitudes & practices of breastfeeding** Mixed feeding ++++^B^ / Benefits of breastfeeding +++^E^	Although there were no statistically significant associations between any of the socio-demographic factors and EBF, in all themes except **Mothers’ knowledge, attitudes & practices of breastfeeding**, mothers mentioned and discussed barriers much more than enablers of EBF. Furthermore, as an infant feeding practice, the high prevalence of mixed feeding was supported by the qualitative data with the code Mixed feeding having emerged as the only infant feeding description and topic of discussion. EBF itself did not emerge as a code but was discussed in relation to the benefits of breastfeeding, information on infant feeding, and in relation to the frequency and duration of breastfeeding. The three barrier codes were Mixed feeding, Mother’s stress and Home environment as explained by the quotes below
	**Family environment**	
	Home setting ++++^B^ / Home setting +++^E^ **Social environment**	
	Public spaces & places (malls, taxis, other people)+++^B^ / Health professionals++^E^ **Baby cues** Baby’s stomach ailments++^B^ / Baby’s Health ++^E^	
**Supporting quotes for mixed feeding from mothers by dominant codes (F = FGD number: M = Mother number)**		
Mixed feeding ++++^B^		
‘*I realised that when I feed the baby formula, the baby was getting full and gained weight but not with breastmilk. Then I decided to only give formula and stop breastfeeding’.-* 27 years old, 17 week old baby has three children (F3:M1)		
Mother’s stress ++++^B^		
‘*Let me say, maybe I am stressing about something, I can’t breastfeed with my high level of stress because it can cause problems for the baby like diarrhoea for the baby. My breastmilk is not okay (for the baby). Even though the baby cries, I must try to reduce the level of stress before I breastfeed so that it doesn’t affect the baby. That’s why they say, if you are breastfeeding, do it with love. Love your baby.’* –38 years old with three children (F1: M3)		
Home environment ++++^B^		
‘*Or maybe she is angry, her man is not around to assist with the baby and the baby doesn’t want to or struggles to suck at her breast’.-* 26 years old, 18 week old baby, has 3 children – (F2:M4)		
**EBF practice**	**Themes and codes by frequency counts for barrier (B) and enabler (E)**	**Interpretation of findings**
EBF rates with infant age EBF 4–8 weeks: 34.0% EBF 10–14 weeks: 15.1% EBF 20–24 weeks: 9.1%	**Mothers’ knowledge, attitudes & practices of breastfeeding** Benefits of breastfeeding +++^E^ **Family environment** Home setting +++^E^ **Mothers’ attributes: physical and mental wellbeing, experience and relationships** Mother’s positive emotions++^E^ **Social environment** Health professionals++^E^ **Baby cues** Baby’s health ++^E^	EBF decreased significantly with infant age with the highest EBF rate at 4–8 weeks. This may be explained by the increased contact with health services during ANC and the first 6-weeks post-partum during which BF support and promotion is the main focus of post-natal care of the infant. This high rate of EBF at 4–8 weeks was supported by the codes Benefits of breastfeeding, Baby health and Health professionals. Illustrating the positive influence of the health services on EBF. The codes Home setting, Mother’s positive emotions and Health professionals were reflected in the high BSES scores (85.6%). The BSES domain of physiological and affective states infers that positive interpretations from cues, support BF such as support and encouragement from family, health professionals or a positive interaction with the infant such as Baby’s health.
**Supporting quotes from mothers by dominant codes (F = FGD number: M = Mother)**		
Benefits of breastfeeding +++^E^		
*‘At the clinic, they said that mixed feeding - both formula and breast milk - the baby can have a reaction. As you go out to the clinic, the baby wants to feed and you don’t have anywhere to warmth up the formula bottle and you don’t know where and the breast milk it’s always warm and that’s how we end up having frequently sick babies. So I think breast feeding is much better.’-.26 years old, 14 week old baby, mother of three children* (F4:M2)		
Home setting +++^E^		
*‘Because if you get more support from the family then you will also feel that you should keep breastfeeding the baby. Breastfeed and breastfeed. Now and then you feel like you are not getting support from the family then you say to yourself, I will just leave this baby here. Then I am going to leave and I am not going to give the child my breast.’*- 24 years with 2 children (F6:M3)		
Mother’s positive emotions++^E^		
‘*you just become happy when you breastfeed. You are happy all the time. Anyway, circumstances make us give our babies formula milk. It’s like with me or maybe it was because I was a first time mother and I was happy about having a baby’*- 33 years with three children (F3:M4)		
Health professionals++^E^		
‘*I told the nurse that I didn’t know what was going on and that’s when she showed me how to breastfeed the baby’*- 21 years old with first baby (F5:M1)		
**Screening measures**	**Themes and codes by frequency counts for barrier (B) and enabler (B)**	**Interpretation of findings**
Edinburgh Postnatal Depression Scale (EPDS) score EPDS < 10: 74 (55.2) EPDS ≥10: 60 (44.8) EPDS score was not statistically associated with breastfeeding practices	**Mothers’ attributes: physical and mental wellbeing, experience and relationships** Mother’s stress ++++^B^ **Mothers’ knowledge, attitudes & practices of breastfeeding** Mixed feeding ++++^B^ **Home environment** Home setting ++++^B^ **Social environment** Public spaces & places (malls, taxis, other people)+++^B^ **Baby cues** Baby’s stomach ailments++^B^	The unexpected high prevalence of postnatal depression (44.8%) found amongst mothers is reflected in higher number of barriers in most of the themes. Furthermore, it is understandable and reasonable that mothers who are in a negative mental disposition would be distressed. This high level of negative mental disposition was reflected in the code Mother’s stress. The codes Home setting, Public spaces and places and Baby’s stomach ailments illustrate the sources of Mother’s stress. Baby’s stomach ailments support and explained the use of inappropriate Traditional beliefs & practices which led to the high prevalence of Mixed feeding. Low breastmilk supply is understood by mothers to cause Baby’s stomach ailments and in turn mothers mix fed and gave their babies traditional medicines to remedy the situation. Mothers’ stress was also understood to affect the baby emotional state and caused an unhappy baby. Mother’s believed that their negative emotions could be felt and carried over to their babies through their breastmilk.
Mother’s stress++++^B^		
*It gives me stress (laughs) you will be instructed to eat soft-porridge while staring at them (family) eating a decent plate (of food). By that time you are been told that you will feed the baby unhealthy milk, and remember that porridge it’s like water it won’t make you full and does not have all the nutrients required* (for making breastmilk).- 26 years old, 14 week old baby, mother of three children (F4:M2)		
Family environment ++++^B^		
*When you are not in a good home environment, you are always angry, you get mood swings and sometimes you*		
*lose appetite, so you don’t eat and the baby suffers to get enough breastmilk from you.* -29 years old first time mother, baby 6 weeks old (F1:M5)		
Social environment+++^B^		
*We are giving our babies the Muthi wenyoni and Qhuma (traditional medicines) because that’s how we were raised. For us, it is easy to consider those (medicines) and give them to our babies to drink. We are grown up by elderly people who advise us. It is their advice that we must buy our children those medicines.-* 33 years old, 23 week old baby, mother of three children (F4:M4)		
**Screening measures**	**Dominant codes**	**Interpretation of findings**
Breastfeeding Self-Efficacy Score- Short-Form BSES < 60: 27 (19.4%) BSES ≥60: 112 (80.6%) BSES-SF score was not statistically associated with breastfeeding practices	**Mothers’ knowledge, attitudes & practices of breastfeeding** Benefits of breastfeeding +++^E^ **Home environment** Home setting +++^E^ **Mothers’ attributes: physical and mental wellbeing, experience and relationships** Mother’s positive emotions++^E^ **Social environment** Health professionals ++^E^ **Baby cues** Baby’s Health ++^E^	Despite the prevalence of high breastfeeding efficacy (80.6%) at 4–8 weeks, EBF prevalence was low (34.0%). For those mothers EBF, motivators were Benefits of breastfeeding, and Baby’s health, support in the home setting and from health professionals. Positive emotional cues and reinforcement of Mother’s positive emotions supported EBF.
Benefits of breastfeeding+++^E^		
*Healthy baby doesn’t get affected from anything because the baby only breastfeeds from the mother. The baby doesn’t get affected by any infection or any disease complications because the baby only gets breastmilk.*- 26 years old, baby 18-week, mother of two children (F1:M4)		
Home environment+++^E^		
*So when you get the support at home from your husband or family members, you can breastfeed easily. When you don’t get that support in most cases, you won’t easily breastfeed like a stress-free mother.* - 38 years old, 10-week old baby, mother of three children (F3:M3)		
Mother’s positive emotion++^E^		
‘*Breastfeeding as a happy smiling mom means the breastmilk its more healthier than when you are angry, … breast feeding with love, enjoying what she is doing (breastfeeding)’ – 22 year old, first time mother, 7-week old baby (F6:M4)*		

As seen in Table 1, the majority of mothers were unmarried (84.9%), living with family (69.2%) and unemployed (74.2%). This is supported by the barrier codes *mother’s stress* and *home setting* as illustrated by this focus group participant in response to what makes breastfeeding difficult? *‘Financial support. As sometimes the absence of the father, you delivered a baby who is fatherless. You think what am I going to eat so that I can breastfeed? How will I provide for the child? And that makes you stop breastfeeding to feed the baby some rooibos (*tea)’ *–* unemployed, 29 year-old, first time mother.

A high percentage of mothers had high school education or post-high school training (93.9%), but were unemployed (74.2%) and the majority of mothers (80.6%) scored high on the BSES-SF (≥ 60), but also a higher than expected proportion of mothers (44.8%) had scores on the EPDS indicating possible presence of depression symptoms (≥ 10) as shown in Table 1. *Mother’s stress*, *home setting* and *relationship with the father of the child* are barrier codes as illustrated by this FGD participant, ‘*Sometimes when it’s tough and you are full of stress, you think of going job hunting. I wasn’t interested in breastfeeding. I remember when the baby was newly born, I had nothing, not even baby’s nappies. I was even thinking of giving the baby to the baby’s father because I was stressed and had nothing. Even my mind was not committed to breastfeeding because I couldn’t cope anymore’ –* unemployed 33 year-old mother with three children.

A third of mothers were not able to report on their household monthly income (30.2%), while only a little more than half of the cohort (55.3%) reported a household income of more than US$200/month. Financial demands are high and food is a major concern for mothers as explained by this participant, *‘I also think that it’s the support at home. Yes, especially regarding food, you can’t breastfeed while you are hungry. Then you have to make do with food like soft (maize) porridge’* - 24 year-old domestic worker with two children. This financial strain and focus on food is reflected in the barrier codes *mother’s stress, mother’s health status and physical wellbeing, mother’s hunger and nutrition* and *food at home*.

At 4–8 weeks, over two-thirds of the cohort used conventional non-prescribed medicines for their infants (67.3%). These practices are supported and encouraged by the elders in the family as explained by this participant *‘We follow the rules and the culture as we are growing up and the grown-ups will say we were using those* (medicines) *on you, when you were a baby and as you were growing up. So why now should you want to follow the western ways? We just follow the wisdom of our grown-ups’* – 36 year old employed mother with three children. The codes *conventional medicines for babies, advice from elders* and *traditional beliefs and practices* were barriers to breastfeeding.

The decrease in EBF with infant age from 34.0% at 4–8 weeks to 18.0% at 20–24 weeks shown in Table 2 is eloquently explained by this mother: *‘Can I just be honest, the reasons why we don’t manage* (to EBF)*, when they are still infants around 7-10 days they get full enough of just been breastfed. The bigger the baby gets the more the intestines grow so you won’t manage only with breastfeeding. They want something that will last longer in their stomach’* - 34 years old, employed mother of three children. The codes *mother’s perception of breastmilk supply, foods to make or increase breastmilk supply* and *baby not full* were barriers to breastfeeding.

The main disrupter of EBF was providing water to the infants, with 39.4% of 4–8 week olds already receiving water. Of these infants 28.7% received water with added sugar. As explained by this mother, *‘When you breastfeed a baby and mix with some sugar-water, the baby becomes full and the baby doesn’t cry when you don’t have enough (breast) milk’ – 29 years old, with two children.* The codes *mixed feeding, mother’s perception of breastmilk supply, advice from elders* and *baby stomach ailments* were barriers to breastfeeding.

Mixed feeding with infant formula increased with age with 17% mixed feeding with formula at 4–8 weeks to 30.6% at 20–24 week. Reasons for mixed feeding with formula is explained by this mother. *‘Speaking for myself, I started with just breastfeeding but because I didn’t have much time and also to give the baby more attention as I am a working mom and I have an older kid then I came to a decision that I should give (formula) milk. I also couldn’t produce a lot of breastmilk which required me to always be close to him to breastfeed him, which I couldn’t do because I didn’t have enough (breast) milk’* – 24 year-old with two children. The codes *mixed feeding, mother’s perception of breastmilk supply* and *expressing breastmilk* supported the formula feeding practice.

Food feeding was 3.1% at 4–8 weeks with a three-fold increase at 10–14 weeks (9.4%) which more than doubled by 20–24 weeks (20.0%). This pattern of mixed feeding is consistent with other studies conducted across South Africa [42, 46–48]. As explained by this mother, ‘*If the baby consumes more then you are able to produce milk. Then you know you can’t produce more (milk), it’s where now you reach a decision that you will end up giving those cereals so that the baby can get full’ –* 24 years old, two children*.* The codes *mixed feeding, mother’s perception of breastmilk supply* and *baby not full* supported premature food feeding to infants aged 4–24 weeks.

Table 5 shows that in the theme of Mother’s knowledge, attitudes & practices of breastfeeding, the code *mixed feeding* had the highest frequency count and reflects in the dominant infant feeding practice of the cohort. The code *mothers’ stress* had the highest frequency count of all the codes highlighting Mother’s attributes (physical and mental wellbeing, experience and relationships) as the dominant theme. Sources of mother’s stress related to mother’s perception of breastmilk supply, access to food and regular meals in the home and difficult relationships in the home, highlighting the difficult lived experience of breastfeeding mother’s in low-income households.

## Discussion

This cohort of mothers had low prevalence of EBF and high prevalence of mixed feeding which reflects the findings of many other studies in South Africa [46–48]. There was a significant drop in EBF prevalence from 4 to 8 weeks to 20–24 weeks which corresponded with the increasing prevalence of formula milk and food feeding over time, with half of all infants respectively getting formula and/or food. This pattern of suboptimal breastfeeding of infants was reflected in mothers discussing more barriers than enablers of EBF during the FGDs. Furthermore, *mixed feeding* was a dominant code in the theme Mother’s knowledge, attitudes & practices of breastfeeding.

The findings of the qualitative data highlighted five main themes, Mothers’ attributes: physical and mental wellbeing, experience and relationships, Mothers’ knowledge, attitudes & practices of breastfeeding, Family environment, Social environment and Baby cues. These themes are in line with other research on the ecological framework of breastfeeding [49] which has been further expanded by the model for the determinants of breastfeeding [8] which recognizes the mother-infant dyad, family and home setting and the broader social environment.

Of all the themes, the code *mothers stress* was the single highest scoring code and reflects the immense and difficult circumstances mothers from low-income households are faced with in general, but particularly as breastfeeding mothers. The intersectionality of inequity and poverty for mothers was expressed by mothers as experiences of stress and at times distress. The discussions revolved around the unsupportive home environment which was supported by the finding that the majority of mothers lived with their families rather than with their partners or spouses.

South Africa has a high number of single mothers with just over 60% of children born in 2017 not having a registered father [50]. Furthermore, given the general high levels of poverty in South African townships and the high levels of unemployment amongst mothers in the cohort study, a major concern and source of stress for breastfeeding mothers was the lack of food in the home. In 2017, almost 20% of South African households had inadequate or severely inadequate access to food, with the North West province having the highest number of food insecure households at 63% [36].

Both family stress and the lack of food to support breastfeeding, affected mothers’ mental health. This negative mental disposition was reflected in the high EPDS scores compared to global norms. A meta-analysis showed that about 13% of mothers in developing countries experience clinical depression after childbirth [51]. WHO further asserts that the global prevalence is much higher than this figure which was derived from research conducted mostly from developed countries [52]. Mental disposition among breastfeeding women can be measured as postnatal depression [53]. Women with high EPDS scores have been found to be more likely to stop breastfeeding within 3 months [54]. However, in the context of South Africa, because of financial constraints mothers are less likely to stop breastfeeding completely but are more likely to mix feed their infants as reflected in the most recent DHS [46]. In the current study, EPDS scores at 4–8 weeks postpartum had no association with EBF or exclusive formula feeding.

Despite the evidence of limited maternal nutrition impact on breastmilk supply and quality [52], mothers perceived and internalized that the stress in the home and the lack of food negatively affected their mental disposition and in turn negatively impacted on their ability to produce sufficient breastmilk of good quality for her infant [53]. Public health interventions to support breastfeeding also counsel mothers on nutrition during pregnancy and lactation. In a study, 84% of mothers had knowledge that diet should be changed by increasing, adding or avoiding some special food items in the diet during pregnancy and lactation [54].

In our study, mothers were acutely aware that what they eat or do not eat will affect their ability to produce breastmilk and the quality of their breastmilk. They were also able to name foods that they believed or were told would improve breastmilk production. Nutrition during pregnancy and lactation has opened a multi-billion-dollar industry for nutrition supplements and supplemental feeds for pregnant and breastfeeding mothers. Mothers are also bombarded by food and nutrition guidance from family, relatives and health professionals as illustrated by this quote*, ‘If after birth, you are under your mom’s care it becomes difficult because you get instructed, “you will drink coffee and eat your soft porridge” always, and they don’t give you fatty food before the umbilical cord is healed’.*

Furthermore, aggressive marketing of infant formula has exploited this understanding that a mother’s nutrition affects her quality of breastmilk and what she is able to provide her breastfeeding baby, as graphically illustrated in a formula industry funded marketing campaign in Brazil on ‘*Your child is what you eat. Your habits in the first thousand days of gestation can prevent your child from developing serious diseases’* [55]*.* Mothers’ decisions to mix feed their infants are in response to a number of Baby cues that the mother interprets that the baby is not getting enough breastmilk. Mothers interpret and internalize these Baby cues as signalling that their breastmilk is not good enough in quantity or quality and therefore an alternative solution is needed. This solution, more often than not, is to complement breastfeeding with infant formula and food.

Though EBF was low, there were enablers identified in each of the themes. Of all the themes, only the codes *benefits of breastfeeding* in the theme Mother’s knowledge, attitudes & practices of breastfeeding and access to and information and/or services from *health professionals* in the theme Social environment were stronger enablers compared to the barriers identified in those themes. With more than 75% of public health facilities accredited as baby-friendly [56], over 95% of mothers delivering in a health facility [46] and 90% registered on the national mHealth platform [57], mothers knowledge, attitudes of breastfeeding should have been well established. The fact that mixed feeding is a norm reflects on the disempowering and hostile environments breastfeeding mothers are confronted with [58, 59]. Hence the number of global and national initiatives to improve the breastfeeding environments with specific focus on addressing psychosocial barriers to breastfeeding [25, 60, 61].

Other enablers identified were the codes, *mother’s positive emotion (happy, feels good)* in the theme Mothers attributes, support in the *home setting* in the theme Family environment and *baby’s health* in the theme Baby cues. This was also supported in the relatively high BSES score of the cohort. BSES is influenced by four main sources of information: (1) performance accomplishments (e.g., past breastfeeding experiences); (2) vicarious experiences (e.g., watching other women breastfeed, seeing breastfeeding in public spaces); (3) verbal persuasion (e.g., encouragement from influential others such as friends, family, and health professionals); and (4) physiological responses (e.g., fatigue, stress, anxiety). In each of these domains, the cohort had positive features with trends of higher BSES scores with higher parity, with high breastfeeding practices, regular access to breastfeeding information and contact with breastfeeding promoting health professionals and their positive disposition towards breastfeeding.

While BSES has demonstrated to predict EBF in other settings [62–67], this was not the case for this cohort. Unlike other settings this cohort displayed both high BSES scores and high EPDS scores, which may be explained by South African’s chronic stressful environments, but generally, mothers’ strong coping mechanisms and resilience to shocks and insults at the individual and societal level [51, 68, 69].

Mothers have mentioned both the Family and Social environment more often as barriers than as enablers of exclusive breastfeeding. In the context of our research setting, the lived realities of low-income households are plagued with food insecurity, hardship and strife [36, 69, 70]. Exclusive breastfeeding is an additional burden on an unsupported, unemployed breastfeeding mother who is physically the sole provider for the health and wellbeing of herself and her infant. In the Family environment the lack of support from family to assist the breastfeeding mother with household chores and family members’ negative interventions when there are breastfeeding difficulties were reported. Furthermore, mothers experienced the social environment as hostile with negative judgements of breastfeeding, or high expectations of breastfeeding mothers from complete strangers or from society at large with no or few facilities to support breastfeeding mothers in public spaces and places like shopping malls, restaurants, and public institutions. This is supported by the efforts of civil society to normalize breastfeeding in public spaces [59, 71, 72].

With the majority of mothers being unemployed, the work setting did not emerge as a strong theme and is captured within the theme Social environment. While the health setting did emerge as an enabling factor, it was limited to the mother’s encounter with health professionals at the health clinic or hospital. This was not unexpected as South Africa has a high coverage of primary healthcare facilities and high utilization rates especially for antenatal care and child health services [46]. Additionally, South Africa has a very well established and a 25-year history of the Baby Friendly hospital initiative which has significantly scaled up in coverage since 2011 [55]. In recent times, South Africa has implemented at scale mHealth services to pregnant women and mothers of infants through cell phone based health messaging [11, 73], increasing the reach and intensity of health service-driven breastfeeding messaging to mothers.

The lower emphasis on the theme Baby cues is supported by literature that responsive parenting skills and identification and appropriate response to baby needs is lacking [74]. The South African National Department of Health has responded to this need with the revised road-to-health booklet which is in line with the nurturing care framework that focuses on five pillars namely, nutrition, love, protection, healthcare and extra care [75]. Mothers interpret and internalize baby crying, baby breastfeeding frequently and baby stomach ailments like cramps, burps, and not passing stools as signals that their breastmilk is not enough or is not of good quality to satisfy their infants’ nutritional needs. While the mother’s decision to introduce other foods to her baby may silence the negative Baby cues and soothe the mother and the household, scientific research has established that infants who are mixed fed have poorer health and development outcomes than EBF infants [7]. Literature has correctly reported that there is a gap in proven effective interventions that are delivered at the household level [9]. Furthermore, in the South African context, studies using breastfeeding education, peer support and counselling have not rendered the desired EBF outcomes [39, 74]. This calls for a redesign of breastfeeding support programmes that will adequately and appropriately address the psychosocial barriers as articulated by mothers themselves.

## Conclusions

The breastfeeding patterns of this cohort study are similar with other South African studies with low EBF rates and high rates of mixed feeding. Despite the EPDS data showing that a relatively high proportion of mothers experienced possible presence of postnatal depression symptoms, the majority of mothers displayed a high level of breastfeeding self-efficacy. This study sought to explore the barriers and enablers of EBF from the perspectives and lived experiences of mothers. The strong emphasis on Mothers’ attributes, rather than on Family environment, or the Social environment demonstrates that the objective to capture the mothers’ lived experiences was met. The low emphasis and the focus on the negative of Baby cues is worrisome as the healthy mother-infant dyad is imperative for optimal health outcomes of mothers and infants.

The qualitative data revealed that breastfeeding mothers from low-income households experience high levels of stress which they believed undermined their ability to produce enough breastmilk and to produce breastmilk of good quality for their infants. Mothers interpreted and internalized infant cues as negative responses to their breastmilk. Baby crying, baby breastfeeding frequently and not sleeping long periods were interpreted as signals of not enough breastmilk. These seem to be the main drivers for mothers’ decisions to mix feed their infants. If South Africa is to reach the global nutrition goal of 50% EBF by 2025 and reap the full benefits of EBF, interventions to support breastfeeding mothers to optimally EBF should be explored, designed and implemented. These interventions should address food insecurity and family relations as well as help build confidence and resilience in mothers who are confronted by difficult environments in the home and broader society.

## Data Availability

The dataset or transcripts are available from the corresponding author on reasonable request.

## Abbreviations

BF: Breastfeeding
BSES-SF: Breastfeeding Self-Efficacy Scale – Short Form
EBF: Exclusive breastfeeding
EPDS: Edinburgh Postnatal Depression Scale
FGDs: Focus Group Discussions

## References

[1] World Health Organization and UNICEF (2014). Global Nutrition Targets 2025: Breastfeeding policy brief.

[2] World Health Organization and UNICEF (2015). Breastfeeding Advocacy Initiative.

[3] Kakietek J, Eberwein JD, Walters D, Shekar M (2017). Unleashing gains in economic productivity with investments in nutrition.

[4] Kramer MS, Kakuma R (2012). Optimal duration of exclusive breastfeeding. Cochrane Database Syst Rev.

[5] Victora C, Bahl R, Barros A, França G, Horton S, Krasevec J, Murch S (2016). Breastfeeding in the 21^st^ century: epidemiology, mechanisms, and lifelong effect. Lancet.

[6] World Health Organization and UNICEF (2003). Global strategy for infant and young child feeding.

[7] Sankar MJ, Sinha B, Chowdhury R, Bhandari N, Taneja S, Martines J (2015). Optimal breastfeeding practices and infant and child mortality: a systematic review and meta-analysis. Acta Paediatr.

[8] Rollins NC, Bhandari N, Hajeebhoy N, Horton S, Lutter CK, Martines JC (2016). Why invest, and what it will take to improve breastfeeding practices?. Lancet.

[9] Sinha B, Chowdhury U, Taneja S, Jose M, Bahl R, Sankar MJ (2017). Integrated interventions delivered in health systems, home, and community have the highest impact on breastfeeding outcomes in low- and middle-income countries. J Nutr.

[10] Oriá MOB, Dodou HD, Chaves AFL, Santos LMDA, Ximenes LB, Vasconcelos CTM (2018). Effectiveness of educational interventions conducted by telephone to promote breastfeeding: a systematic review of the literature. Rev Esc Enferm USP.

[11] Lee SH, Nurmatov UB, Nwaru BI, Mukherjee M, Grant L, Pagliari C (2016). Effectiveness of mHealth interventions for maternal, newborn and child health in low- and middle-income countries: systematic review and meta-analysis. J Glob Health.

[12] Afoakwah G, Smyth R, Lavender DT (2013). Women’s experiences of breastfeeding: a narrative review of qualitative studies. African J Midwifery Women’s Health.

[13] Bai YK, Middlestadt SE, Joanne Peng CY, Fly AD (2009). Psychosocial factors underlying the mother’s decision to continue exclusive breastfeeding for 6 months: an elicitation study. J Human Nutr Dietetics.

[14] Raman S, Nicholls R, Ritchie J, Razee H, Shafiee S (2016). Eating soup with nails of pig: thematic synthesis of the qualitative literature on cultural practices and beliefs influencing perinatal nutrition in low and middle income countries. BMC Pregnancy Childbirth.

[15] Balogun OO, Dagvadorj A, Anigo KM, Ota E, Satoshi S (2015). Factors influencing breastfeeding exclusivity during the first 6 months of life in developing countries: a quantitative and qualitative systematic review. Maternal Child Nutr.

[16] UNICEF (2016). Breastfeeding and the sustainable development goals.

[17] World Health Organization, UNICEF (2017). Tracking progress for breastfeeding policies and programmes: Global breastfeeding scorecard 2017.

[18] Dias CC, Figueiredo B (2015). Breastfeeding and depression: a systematic review of the literature. J Affect Disord.

[19] Norhayati MN, Nik Hazlina NH, Asrenee AR, Wan Emilin WMA (2015). Magnitude and risk factors for postpartum symptoms: a literature review. J Affect Disord.

[20] McCarter-Spaulding D, Gore R (2009). Breastfeeding self-efficacy in women of African descent. J Obstet Gynecol Neonatal Nurs.

[21] Otsuka K, Dennis C-L, Tatsuoka H, Jimba M (2008). The relationship between breastfeeding self-efficacy and perceived insufficient milk among Japanese mothers. J Obstet Gynecol Neonatal Nurs.

[22] Risenga L (2014). Lived experience of first time mothers towards breastfeeding at Muyexe village in Mopane District, Limpopo Province. J Med Medical Res.

[23] Naidoo K (2008). Researching reproduction: reflections on qualitative methodology in a transforming society. Forum Qualitative Sozialforschung / Forum: Qualitative Social Research.

[24] Goosen C, McLachlan M, Schubl C (2014). Factors impeding exclusive breastfeeding in a low-income area of the Western Cape Province of South Africa. Africa J Nurs Midwifery.

[25] Menon P, Nguyen PH, Saha KK, Khaled A, Kennedy A, Tran LM (2016). Impacts on breastfeeding practices of at-scale strategies that combine intensive interpersonal counseling, mass media, and community mobilization: results of cluster-randomized program evaluations in Bangladesh and Viet Nam. PLoS Med.

[26] Cresswell JA, Ganaba R, Sarrassat S, Somé H, Diallo AH, Cousens S (2019). The effect of the Alive & Thrive initiative on exclusive breastfeeding in rural Burkina Faso: a repeated cross-sectional cluster randomised controlled trial. Lancet Glob Health.

[27] Upton J, Gellman MD, Turner JR (2013). Psychosocial Factors. Encyclopedia of Behavioral Medicine.

[28] Department of Health (2013). Infant and young child feeding policy.

[29] National Department of Health (2017). Circular minute number 3 of 2017/18 HIV/AIDS, TB, MNCWH: amendment of the 2013 infant and young child feeding (IYCF) policy.

[30] Department of Health (2016). National breastfeeding campaign overview South Africa (2016–2017).

[31] Department of Health (2018). What you should know about breastfeeding.

[32] South Africa. Department of Health (2012). Foodstuffs, Cosmetics And Disinfectants Act, 1972 (Act No. 54 of 1972). Regulations Relating To Foodstuffs For Infants And Young Children. Government Gazette No. 35941:991.

[33] Department of Health (2011). Implementation plan for breastfeeding promotion in South Africa.

[34] Kapilashrami A, Hill S, Meer N (2015). What can health inequalities researchers learn from an intersectionality perspective? Understanding social dynamics with an inter-categorical approach?. Soc Theory Health.

[35] Stastics South Africa (2019). Quarterly Labour Force Survey.

[36] Statistics South Africa (2018). General Household Survey, 2017.

[37] Creswell JW, Plano Clark VL (2011). Choosing mixed methods research design. In designing and conducting mixed methods research: SAGE.

[38] Guest G, Bunce A, Johnson L (2006). How many interviews are enough?: an experiment with data saturation and variability. Field Methods.

[39] Tylleskar T, Jackson D, Meda N, Engebretsen I, Chopra M, Diallo A, Doherty T (2011). Exclusive breastfeeding promotion by peer counsellors in sub-Saharan Africa (PROMISE-EBF): a cluster randomized trial. Lancet.

[40] Shisana O, Labadarios D, Rehle T, Simbayi L, Zuma K, Dhansay A, Reddy P, Parker W, Hoosain E, Naidoo P, Hongoro C, Mchiza Z, Steyn NP, Dwane N, Makoae M, Maluleke T, Ramlagan S, Zungu N, Evans MG, Jacobs L, Faber M, the SANHANES-1 Team (2014). South African National Health and nutrition examination survey (SANHANES-1).

[41] World Health Organization (2008). Indicators for assessing infant and young child feeding practices: conclusions of a consensus meeting held 6–8 November 2007 in Washington D.C., USA.

[42] Goosen C, McLachlan M, Schubl C (2014). Infant feeding practices during the first 6 months of life in a low-income area of the Western Cape Province. South African J Child Health.

[43] Cox JL, Holden JM, Sagovsky R (1987). Detection of postnatal depression: development of the 10-item Edinburgh postnatal depression scale. Br J Psychiatry.

[44] Mokwena K, Shiba D (2014). Prevalence of postnatal depression symptoms in a primary health care clinic in Pretoria, South Africa: management of health care services. African J Physical Health Education Recreation Dance.

[45] Rothman M, Berti C, Smuts CM, Faber M, Covic N (2015). Acceptability of novel small-quantity lipid-based nutrient supplements for complementary feeding in a peri-urban south African community. Food Nutr Bull.

[46] National Department of Health, Statisics South Africa (Stats SA), South African Medical Council (SAMRC), ICF (2017). South African demographic and health survey, 2017: Key indicators.

[47] Budree S, Goddard E, Brittain K, Cader S, Myer L, Zar HJ (2017). Infant feeding practices in a south African birth cohort — a longitudinal study. Maternal Child Nutr.

[48] Horwood C, Haskins L, Engebretsen IM, Phakathi S, Connolly C, Coutsoudis A (2018). Improved rates of exclusive breastfeeding at 14 weeks of age in KwaZulu Natal, South Africa: what are the challenges now?. BMC Public Health.

[49] Tiedje LB, Schiffman R, Omar M, Wright J, Buzzitta C, McCann A, Metzger S (2002). An ecological approach to breastfeeding. Am J Maternal Child Nurs.

[50] Hall K, Richter L, Mokomane Z, Lake L (2018). South African child gauge 2018.

[51] O'Hara MW, Swain AM (1996). Rates and risk of postpartum depression—a meta-analysis. Int Rev Psychiatry.

[52] World Health Organization (WHO) (2009). Mental health aspects of women’s reproductive health: A global review of the literature.

[53] O’Brien M, Buikstra E, Hegney D (2008). The influence of psychological factors on breastfeeding duration. J Adv Nurs.

[54] Bick DE, MacArthur C, Lancashire RJ (1998). What influences the uptake and early cessation of breast feeding?. Midwifery.

[55] Innis SM (2014). Impact of maternal diet on human milk composition and neurological development of infants. Am J Clin Nutr.

[56] Hutchinson AD, Charters M, Prichard I, Fletcher C, Wilson C (2017). Understanding maternal dietary choices during pregnancy: the role of social norms and mindful eating. Appetite.

[57] Mahmood S, Atif M, Mujeeb SS, Bano N, Mubasher H (1997). Assessment of nutritional beliefs and practices in pregnant and lactating mothers in an urban and rural area of Pakistan. J Pakistan Med Assoc.

[58] UK BMA. Nestle-sponsored paediatric society shocks breastfeeding mothers with Brazilian first 1000 days campaign according to report in the Daily Mail 2015. [http://www.babymilkaction.org/archives/6899] Accessed on 18 May 2020.

[59] Martin-Wiesner P (2018). A policy-friendly environment for breastfeeding: a review of South Africa’s progress in systematising its international and national responsibilities to protect, promote and support breastfeeding.

[60] Seebregts C, Dane P, Parsons AN, Fogwill T, Rogers D, Bekker M (2018). Designing for scale: optimising the health information system architecture for mobile maternal health messaging in South Africa (MomConnect). BMJ Glob Health.

[61] Brown A (2019). Breastfeeding is not ‘easy’ – stop telling new mothers that it is. The Conversation UK.

[62] Dennis C-L (2003). The breastfeeding self-efficacy scale: psychometric assessment of the short form. J Obstet Gynecol Neonatal Nurs.

[63] Aluş Tokat M, Okumuş H, Dennis C-L (2010). Translation and psychometric assessment of the breast-feeding self-efficacy scale-short form among pregnant and postnatal women in Turkey. Midwifery.

[64] Oliver-Roig A, d’Anglade-González M-L, García-García B, Silva-Tubio J-R, Richart-Martínez M, Dennis C-L (2012). The Spanish version of the breastfeeding self-efficacy scale-short form: reliability and validity assessment. Int J Nurs Stud.

[65] Wutke K, Dennis C-L (2007). The reliability and validity of the polish version of the breastfeeding self-efficacy scale-short form: translation and psychometric assessment. Int J Nurs Stud.

[66] Zubaran C, Foresti K, Schumacher M, Thorell MR, Amoretti A, Müller L (2010). The Portuguese version of the breastfeeding self-efficacy scale — short form. J Hum Lact.

[67] McCarter-Spaulding DE, Dennis C-L (2010). Psychometric testing of the breastfeeding self-efficacy scale-short form in a sample of black women in the United States. Res Nurs Health.

[68] Tomlinson M, Swartz L, Cooper P, Molteno C (2004). Social factors and postpartum depression in Khayelitsha, Cape Town. S Afr J Psychol.

[69] Nor B, Ahlberg BM, Doherty T, Zembe Y, Jackson D, Ekstrom EC (2012). Mother's perceptions and experiences of infant feeding within a community-based peer counselling intervention in South Africa. Maternal Child Nutr.

[70] Reimers P, Israel-Ballard K, Craig M, Spies L, Thior I, Tanser F (2018). A cluster randomised trial to determine the efficacy of the “feeding buddies” programme in improving exclusive breastfeeding rates among HIV-infected women in rural KwaZulu-Natal, South Africa. AIDS Behav.

[71] Richter LM (2016). Why breastfeeding in South Africa still needs champions. The Conversation.

[72] Witten CB (2017). South Africa has made giant strides in breastfeeding. But it’s still taboo in public places. The Conservation.

[73] Statistics South Africa (2013). Social profile of vulnerable groups. 2002–2012.

[74] Savage JS, Hohman EE, Marini ME, Shelly A, Paul IM, Birch LL (2018). INSIGHT responsive parenting intervention and infant feeding practices: randomized clinical trial. Int J Behav Nutr Phys Act.

[75] Bamford L, Martin P, Slemming W, Richter L (2018). The new road to health booklet demands a paradigm shift. South African J Child Health.

